# Happiness and Inflammatory Responses to Acute Stress in People With Type 2 Diabetes

**DOI:** 10.1093/abm/kay039

**Published:** 2018-06-18

**Authors:** Laura Panagi, Lydia Poole, Ruth A Hackett, Andrew Steptoe

**Affiliations:** Research Department of Behavioral Science and Health, University College London, London, UK

**Keywords:** Type 2 diabetes, Happiness, Mental stress, Inflammatory markers, Stress responses

## Abstract

**Background:**

Positive psychological characteristics in people with type 2 diabetes (T2D) are associated with better health and longevity, and one plausible physiological mechanism involves lower markers of inflammation. Positive affect is related to lower basal inflammatory markers and smaller inflammatory responses to acute stress, but this association in people with T2D remains to be examined.

**Purpose:**

To examine the relationship between happiness and inflammatory markers at baseline and in response to acute stress in people with T2D.

**Methods:**

One hundred forty people with T2D took part in laboratory-based stress testing. We aggregated daily happiness ratings over 7 days before stress testing. During the laboratory session, participants underwent two mental stress tasks—the mirror tracing and the Stroop task. Blood was sampled at baseline and post-stress (up to 75 min post-stress) to detect plasma interleukin-6 (IL-6), interleukin-1 receptor antagonist (IL-1Ra), and monocyte chemoattractant protein-1 (MCP-1). Associations between happiness and inflammatory markers and responses were analyzed using multivariable linear regressions.

**Results:**

Greater daily happiness significantly predicted lower baseline and post-stress IL-6 concentrations, and lower baseline MCP-1, after adjusting for covariates. The association between happiness and reduced basal IL-6 maintained after further controlling for daily sadness. We did not find significant associations between daily happiness and inflammatory responses to acute stress. No associations were detected for IL-1Ra.

**Conclusions:**

Happier individuals with T2D have lower inflammatory markers before and after acute stress, albeit independent of stress responsivity. Findings could provide a protective physiological pathway linking daily happiness with better health in people with T2D.

## Teaser

Happier individuals with type 2 diabetes have lower inflammatory factors before and after acute stress. Inflammatory stress responses did not vary between those reporting higher or lower happiness.

Positive affect encompasses feelings that reflect a state of pleasurable engagement with the environment such as happiness, excitement, joy, and contentment [1], and is a component of the broader theoretical concept of subjective well-being [2]. Accumulating evidence acknowledges the protective role of positive affect on physical health, revealing that people who experience more positive affect live healthier and longer lives [3, 4]. The impact of positive psychological characteristics including positive affect, life enjoyment, self-efficacy, and resilience in people with type 2 diabetes (T2D) has also been documented, delineating significant associations with better glycemic control, fewer diabetes complications, and lower risk of cardiac morbidity and mortality [5–7]. The pathways linking positive affect with better health in T2D are unclear, but one plausible mechanism involves lower markers of inflammation [8].

Increased concentration of inflammatory markers reflects activation of the innate immune system following acute stress [9]. Inflammatory markers include a range of cytokines, including interleukin (IL)-6, IL-1, and monocyte chemoattractant protein (MCP)-1. IL-6 is produced by a variety of cell types after stimulation by the pro-inflammatory cytokine IL-1b, and in turn stimulates the synthesis of the acute phase C-reactive protein (CRP) by hepatocytes [9]. The anti-inflammatory agent, IL-1 receptor antagonist (IL-1Ra) inhibits IL-1 action [10], and IL-1 and IL-6 administration can induce IL-1Ra secretion [11–13]. MCP-1 is included in the chemoattractant cytokines family. It is produced after stimulation by other cytokines and is involved in the regulation of migration and infiltration of macrophages [14].

Both basal/resting levels of inflammatory markers and inflammatory responses/changes in levels after acute stress are of importance, as they can be indicative of either high circulating concentrations or dysregulated responses to naturalistic stressors [15]. Laboratory stress testing is a widely used research strategy that can provide insights into inflammatory responses by measuring inflammatory markers before and after acute stress task. A standard stress protocol is used and measures are taken under controlled conditions, reducing the impact of confounders. The focus of this strategy is on changes in markers from baseline to post-task and up to recovery, which could not be detectable if single measures were taken [16, 17]. IL-6, IL-1Ra, and MCP-1 have been found to increase after mental stress tasks in the laboratory [9, 18, 19], although responses are more consistent for IL-6 than the other measures [9, 18].

Heightened circulating inflammatory markers and dysregulated responses to acute stress are both clinically significant, and relevant to diabetes development and prognosis. Meta-analytic results showed that increased circulating IL-6 levels in initially healthy participants predict T2D onset [20]. People with established T2D have higher baseline and post-stress IL-6 levels compared with healthy sample [21, 22]. Increased basal levels in IL-6, IL-1Ra, or MCP-1 are associated with coronary heart disease (CHD [23–26]), which is a macrovascular complication of diabetes [27]. Notably, increased IL-6 in people with T2D has been prospectively associated with macrovascular events and mortality [28]. Larger IL-6 responses to laboratory stress have also been associated with cardiovascular risk factors in a 3-year follow-up study [29].

Positive affect could act protectively upon health and longevity in people with T2D through lower inflammatory markers and/or smaller inflammatory stress responses. In a large study of 2,873 healthy volunteers, positive affect, as assessed by aggregating ecological momentary assessments (EMA) repeatedly over the day, was inversely associated with IL-6 concentrations in women [30]. Moreover, a U.S. nationally representative study involving 969 participants demonstrated a significant relationship between daily positive events over eight consecutive days and reduced basal IL-6 [31]. Similarly, greater decreases in positive affect on stressful days were predictive of elevated IL-6 [32]. Trait positive affect has also been linked to lower stimulated IL-6 levels in a healthy, community sample [33], and lower IL-6 in chronic heart failure patients [34]. Associations between IL-6 and other positive constructs such as purpose in life, positive relationships, and optimism have also been reported [35, 36]. Interestingly, the association between positive affect and reduced IL-6 was maintained after adjustment for depressive symptoms in some studies [30, 34], suggesting that this relationship is not secondary to the absence of negative affect. Less research investigated the association between IL-1Ra and MCP-1 and psychosocial factors. However, elevated MCP-1 has been linked with depressive symptoms [37, 38] and chronic psychosocial stress [39], whereas both MCP-1 and IL-1Ra have been associated with loneliness in women [19].

Positive psychological attributes have also been linked to smaller inflammatory stress responses. In particular, we previously showed that stress responses in fibrinogen, which is another marker of inflammation, were attenuated immediately after task in people reporting greater daily happiness using EMA [40]. Similarly, optimism has been associated with smaller IL-6 responses 2 hr post-task [41]. A more recent study showed that greater increases in negative mood after experimentally induced sedentariness predicted greater IL-6 responses at 45 min post-task [42]. Loneliness has also been associated with larger IL-1Ra increases immediately and up to 45 min post-task in middle-aged women [19].

Considering the lack of research linking positive affect and inflammatory responses in people with T2D, the purpose of this study was to examine the relationship between positive affect and IL-6, IL-1Ra, and MCP-1 at resting and in response to acute stress in people with T2D. In the current study, we assessed happiness as a measure of positive affect. Specifically, we deliberately recorded happiness ratings once-a-day over seven consecutive days to provide an aggregated measure of daily mean happiness as our predictor variable. Previous research indicated that this method shows wider associations with biological stress responses than a retrospective assessment of positive affect over the previous week using a standard mood scale [43]. Aggregated daily measures may provide a more reliable estimate of affect that is not biased by transient moods on a single day, or recall bias, as well as involve a smaller burden for participants compared with EMA-derived measures [40].

Concluding, we hypothesized that happier participants would have lower inflammatory markers at baseline and after stress, and smaller inflammatory stress responses. Our secondary hypothesis was that the relationship between daily happiness and inflammatory markers/responses was independent of daily sadness.

## Method

### Participants

A total of 140 people aged 50 to 75 years with doctor-verified T2D diagnosis participated in laboratory-based experimental stress testing. Participants were recruited from diabetic outpatient and primary care clinics in London between March 2011 and July 2012. People who reported history or previous diagnosis of CHD, inflammatory diseases, allergies, or mood disorders were excluded from the study. Obesity is one of the main risk factors for diabetes [44]; therefore, it was not possible to exclude individuals with obesity from this study. To detect small to moderate effect sizes (δ = 0.32, *p* < .05), we aimed to recruit at least 125 participants. A fully informed written consent was obtained by all participants, and ethical approval was granted by the UK National Research Ethics Service.

All participants were requested to avoid taking anti-inflammatory or antihistamine medication up to 7 days before laboratory session. Participants were also instructed to avoid vigorous exercise and drinking alcohol from the evening before the session and caffeine or smoking for at least 2 hr before stress testing. On the day of testing, participants who reported colds or other infections were rescheduled.

### Procedure

Daily diaries were collected for 7 days before the laboratory session for the measurement of daily happiness. Participants were then tested individually in our light and temperature controlled laboratory, either in the morning or in the afternoon. We first measured height and weight for the body mass index (BMI) calculation. A venous cannula was then inserted and the participant rested for 30 min. During the last 5 min of the resting phase a blood sample was drawn for the measurement of baseline inflammatory markers. We then administered two mental stress tasks for 5 min each. Blood samples were obtained again immediately after the tasks and 45 and 75 minutes later. Other factors measured but not reported in this paper included blood pressure, heart rate, and salivary cortisol [22].

### Measures

#### Predictor variable: Daily mean happiness

We measured daily happiness using ratings made at the end of each day (every evening) for seven consecutive days before the laboratory testing. Participants were asked to report the extent to which they had been feeling happy on that day using a five-point Likert scale from 0 (not at all) to 4 (a lot). We computed an average happiness score over the week as our predictor variable. This method was introduced by Cohen and colleagues [45], and has been used in more recent studies [43]. Mean coefficient of variation over the week was 0.28, suggesting a relatively small variability in data on average.

#### Outcome variables: IL-6, IL-1Ra, and MCP-1

Blood samples were drawn at four time points (baseline, immediately post-task, 45 min post-task, and 75 min post-task) for the measurement of plasma IL-6, IL-1Ra, and MCP-1. Samples were immediately centrifuged at 2,500 rpm for 10 min at room temperature using ethylenediaminetetraacetic acid tubes. Plasma was removed from the tube and aliquoted into 0.5 ml portions and stored at −80° Celsius until batch analysis at a later date. Plasma IL-6 was analyzed with Quantikine high sensitivity two-site enzyme-linked immunosorbent assay (ELISA) from R&D Systems (Oxford, UK). The minimum limit of detection was between 0.016 and 0.110 pg/ml. IL-1Ra and MCP-1 were analyzed in duplicate using fluorescent-labeled capture antibody beads from Millipore (Milliplex Human Cytokine/Chemokine kit, Millipore Corporation, US), and concentrations were measured using Luminex flow cytometer technology from Bio Rad (Bio-Plex, Hercules, CA, USA). The limit of detection for IL-1Ra was 2.3 pg/ml and for MCP-1 was 1.2 pg/ml. The mean intra-assay coefficients of variation for IL-6, IL-1Ra, and MCP-1 were 7.3%, 4.6%, and 6.1%, respectively. Then mean inter-assay coefficients of variation for the three markers were 7.7%, 6%, and 12% respectively.

#### Covariates

Covariates were selected a priori as previous research indicates that these factors can influence inflammatory activity: age [46], sex [46–48], BMI (kg/m^2^ [49]), smoking (smoker/non-smoker [18]), and household income as an indicator of socioeconomic status (<£20,000, £20,000–40,000, £40,000–60,000, >£60,000 [50]). Age, sex, smoking status, and household income were recorded by self-report. BMI was calculated after measuring height and weight using standardized techniques on the day of testing. Health status is known to influence inflammatory stress responses [9], therefore diabetic medication at the time of testing was selected (oral antidiabetic drugs/insulin or other injectable diabetic medication). Diabetic medication was recorded by self-report and confirmed by inspection of medication packaging on the day of testing. IL-6 shows marked diurnal variation with increasing levels over the course of the day [51]; therefore, time of testing (am or pm) was also selected. In preliminary analysis, we found that happiness showed marginally significant differences between the three marital status groups (single, married, and divorced or separated or widowed as recorded by self-report), and therefore marital status was also included as a covariate. To control for the effects of daily sadness, an average sadness score was recorded by aggregating ratings made at the end of each day for 7 days before laboratory session (same as with daily happiness measurement). Participants were asked to report the extent to which they had been feeling sad on that day using a five-point Likert scale from 0 (not at all) to 4 (a lot). Mean coefficient of variation over the week was 1.18.

#### Other measures

Ethnicity (White, Asian, Afro-Caribbean, and Other), educational level (no qualification/elementary school diploma, up to O levels/middle or junior high school diploma, A-levels-ONC/high school or senior high school diploma, degree/university undergraduate certificate or above), and hours of moderate/vigorous physical activity per week were recorded by self-report. Cardiovascular medication at the time of testing was also recorded by self-report and confirmed by inspection of medication packaging on the day of testing. It was subsequently categorized into anti-hypertensive medication, beta-blockers, cholesterol-lowering drugs, and aspirin. In preliminary analysis, anti-hypertensive medication or beta-blockers use were not significantly associated with baseline inflammatory markers or stress responses, therefore these factors were not included as covariates. Glycated hemoglobin (HbA1c) was assessed from the baseline blood draw using standardized techniques. Subjective stress was measured at baseline, immediately post-task, and 45 and 75 min post-task. We asked participants “how stressed do you feel at the moment” (baseline), “how stressed did you feel during the task” (immediately post-task), and “how stressed do you feel at the moment” (45 and 75 min post-task). Responses were recorded on a seven-point scale, with higher scores indicating greater subjective stress.

### Mental Stress Tasks

Two 5-min standardized mental tasks were administered in random order to induce stress responses, namely an electronic version of the Stroop color-word interference task and the mirror tracing task. These challenges are widely used in experimental studies and have been used in numerous previous investigations by our research group [52, 53]. Briefly, the color-word interference task required successive reporting of target color words (e.g., blue and red) printed in an incongruous color. The mirror tracing task required participants to move a mental stylus to trace a star while looking at the mirror image. When the stylus comes off the star, a mistake is counted by a loud noise emitted by the device (Lafayette Instruments Corp, Lafayette, IN). Participants were informed that the average person can achieve five tracings in the time given.

### Statistical Analysis

We used mean happiness score over a 7-day period as our independent variable and three inflammatory markers (IL-6, IL-1Ra, and MCP-1) as our dependent variables. MCP-1 values were normally distributed, but IL-6 and IL-1Ra were skewed thus were log-n transformed before main analysis. We checked univariate associations between happiness and sample characteristics using Pearson’s *r* correlations for continuous variables, and *t*-tests and one-way between-subjects Analysis of Variance (ANOVA) for categorical variables. We also checked associations between baseline inflammatory markers and their patterns of responsivity using Pearson’s *r* correlations. To test for the effects of stress trial on subjective stress and inflammatory levels, subjective stress ratings, and inflammatory markers were examined across four time points: baseline, immediately post-task, 45 min, and 75 min post-task using one-way repeated-measures ANOVA.

The association between happiness and inflammatory markers was analyzed using multivariable linear regressions on baseline IL-6, IL-1Ra, and MCP-1 values as well as regressions on the post-task values (immediately post-task, 45 min post-task, and 75 min post-task) with all analyses adjusted for age, sex, BMI, smoking, household income, marital status, oral antidiabetic medication, insulin/other injectable diabetic medication, and time of testing. Significant results were further adjusted for daily mean sadness. Separate regression analysis was conducted for each inflammatory marker and at each time point.

The association between happiness and inflammatory stress responses was analyzed using multivariable linear regressions on the mean changes between baseline and post-task values; immediately post-task minus baseline, 45 min post-task minus baseline, and 75 min post-task minus baseline, with analyses adjusted for age, sex, BMI, smoking, household income, marital status, oral antidiabetic medication, insulin/other injectable diabetic medication, and time of testing. Separate regression analysis was run for each inflammatory marker and at each time point. Data from the regression analyses are reported as unstandardized *B* coefficients.

Sensitivity analysis was carried out to check for significant differences in the characteristics of participants between those with full data on inflammatory markers and those with missing data on at least one inflammatory marker. Statistical analyses were conducted using SPSS version 24 (SPSS, Chicago, IL). The level of significance was set at *p* < .05.

## Results

### Sample Characteristics

One hundred forty people with T2D took part in the study. Participants were 63.67 (*SD* = 7.02) years old on average, mostly of White ethnicity, and with relatively low household incomes. The majority of participants held a degree/university undergraduate level certificate or above. The average BMI was within the obese range (BMI > 30 kg/m^2^) and the mean HbA1c was 7.32 % (*SD* = 1.49). The majority of participants were taking oral antidiabetic medication, anti-hypertensive medication, and cholesterol-lowering drugs. Out of 140 participants, 34 participants had missing data for at least one IL-6 measurement, 33 for at least one IL-1Ra measurement, and 34 participants for at least one MCP-1 measurement, owing to issues in blood sampling. More specifically, as the majority of participants were obese, maintaining a functioning cannula for the duration of the session presented technical challenges; the cannula failed part-way through the laboratory procedure for some participants. We had a protocol to not reattempt blood draw in these participants so as to minimize distress. Sensitivity analysis showed that participants of higher BMI were more likely to have missing data on inflammatory markers (*t* = −3.162, *p* = .003, *d* = 0.654), but there were no other differences between those with and without missing data.

Sample characteristics and associations with happiness are presented in Table 1. The mean happiness score was 2.61 (*SD* = 0.87) and was not related to age, sex, BMI, smoking status, medication use, HbA1c, or physical activity. Moreover, no significant differences in happiness were observed for household income, educational level, or ethnicity groups. There were no significant correlations between happiness and subjective stress ratings at baseline or the three time points following stress. The association between happiness and marital status approached significance (*F* = 2.987, *p* = .054, η^2^_p_ = 0.048). We also found a significant negative correlation between happiness and daily mean sadness (*r* = 0.337, *p* < .001).

**Table 1.** Sample characteristics and associations with daily mean happiness (*N* = 122)

Characteristic	*n* (%) or *M* ± *SD*	Associations with happiness^a^: *p* value, effect size
Sex, *n* (%) females	44 (36.1)	*p* = .868, *d* = .034
Age, *M* (*SD*) years	63.67 (7.02)	*p* = .634, *r* = .044
Smoking, *n* (%) smokers	18 (14.8)	*p* = .315, *d* = .263
BMI, *M* (*SD*) kg/m^2^	30.54 (5.59)	*p* = .463, *r* = .067
Marital status, *n* (%)		*p* = .054, η^2^_p_ = .048
Married	62 (50.8)	
Single	26 (21.3)	
Divorced, separated, or widowed	34 (27.9)	
Household income, *n* (%)		*p* = .678, η^2^_p_ = .011
<£20,000	51 (41.8)	
£20,000–40,000	34 (27.9)	
£40,000–60,000	11 (9.0)	
>£60,000	26 (21.3)	
Ethnicity, *n* (%)		*p* = .376, η^2^_p_ = .026
White	97 (79.5)	
Asian	12 (9.8)	
Afro-Caribbean	7 (5.7)	
Other	6 (4.9)	
Educational level^b^, *n* (%)		*p* = .345, η^2^_p_ = .042
No qualifications (elementary school diploma)	10 (8.3)	
Up to O levels (middle or junior high school diploma)	21 (17.5)	
A-levels/ONC (high school or senior high school diploma)	12 (10)	
Degree (university undergraduate certificate) or above	77 (64.2)	
Insulin/other injectable diabetic medication, *n* (%) yes	15 (12.3)	*p* = .661, *d* = .120
Oral antidiabetic medication, *n* (%) yes	98 (80.3)	*p* = .077, *d* = .430
Anti-hypertensive medication, *n* (%) yes	85 (69.7)	*p* = .077, *d* = .330
Beta-blocker, *n* (%) yes	11 (9.0)	*p* = .620, *d* = .193
Cholesterol-lowering drugs, *n* (%) yes	94 (77.0)	*p* = .554, *d* = .117
Aspirin, *n* (%) yes	44 (36.1)	*p* = .557, *d* = .115
Time of testing, *n* (%) am	55 (45.1)	*p* = .576, *d* = .103
HbA1c^c^, *M* (*SD*) %	7.32 (1.49)	*p* = .173, *r* = −.126
Moderate/vigorous physical activity^d^, *M* (*SD*) hours/week	4.61 (8.56)	*p* = .162, *r* = .133
Daily happiness, *M* (*SD*)	2.61 (0.87)	–
Daily sadness^e^, *M* (*SD*)	0.74 (0.73)	*p* ≤ .001, *r* = −.337

*BMI* body mass index; *HbA1c* glycated haemoglobin; *ONC* ordinary national certificate.

^a^Associations between happiness and sample characteristics were tested using Pearson’s *r* correlations for continuous variables, and *t*-tests and one-way between-subjects analysis of variance (ANOVA) for categorical variables; ^b^*n* = 120;^c^*n* = 118; ^d^*n* = 112; ^e^*n* = 120.

We found significant negative correlations between baseline IL-6 and IL-6 responses at the three time points post-task (immediately post-task response: *r* = −0.210, *p* = .018, 45 min response: *r* = −0.266, *p* = .004, 75 min response: *r* = −0.416, *p* < .001), and between baseline MCP-1 and 75 min response (*r* = −0.342, *p* < .001).

## Trial Validity: Subjective Stress and Inflammatory Levels Across Laboratory Session

We found a significant main effect of stress trial for the subjective stress ratings (*F* = 334.786, *p* < .001, η^2^_p_ = 0.717). There were marked increases in subjective stress ratings immediately after the tasks compared with baseline levels (*p* < .001), with values returning to pre-task levels during recovery period.

Significant main effects of trial were also found for IL-6 (*F* = 5.961, *p* = .001, η^2^_p_ = 0.054). IL-6 increased over time, being significantly higher at 75 min post-task compared with baseline (*p* = .021), and immediately post-task values (*p* < .001). The average increase from baseline levels to 75 min was 0.23 pg/ml. No significant effects of stress trial were observed for IL-1Ra (*F* = 0.204, *p* = .876, η^2^_p_ = 0.002). By contrast, MCP-1 showed a progressive decline across the session (*F* = 8.262, *p* < .001, η^2^_p_ = 0.072), so that values at 75 min were significantly lower than baseline (*p* < .001) and immediately post-task (*p* = .003). The average decrease from baseline to 75 min was 7.87 pg/ml. Table 2 presents the subjective stress and inflammatory levels of the participants across the laboratory session.

**Table 2.** Subjective and inflammatory responses to stress

	*n*	Baseline	Immediately post-task	45 min post-task	75 min post-task
Subjective stress	133	1.50^a^ (0.90)	4.50^b^ (1.52)	1.53^a^ (0.96)	1.43^a^ (0.93)
IL-6 (pg/ml)	106	2.08^a^ (1.17)	2.07^a^ (1.11)	2.18 (1.23)	2.31^b^ (1.28)
MCP-1(pg/ml)	107	116.90^a^ (33.84)	115.72^a^ (38.07)	113.08 (36.68)	109.04^b^ (32.15)
IL-1Ra (pg/ml)	106	815.70 (416.84)	815.77 (403.44)	823.28 (415.39)	819.94 (420.77)

Values are presented as means and *SD*. *IL-1Ra* interleukin-1 receptor antagonist; *IL-6* interleukin-6; *MCP-1* monocyte chemoattractant protein-1. Values in rows with different superscripts (^a^, ^b^) are significantly different from one another (*p* < .05).

### Happiness and Inflammatory Markers

#### IL-6

Higher happiness predicted significantly lower plasma IL-6 concentrations for all four time points: baseline (*B* = −0.110, *p* = .043), immediately post-task (*B* = −0.099, *p* = .045), 45 min post-task (*B* = −0.120, *p* = .028), and 75 min post-task (*B* = −0.114, *p* = .033). These effects were independent of age, sex, BMI, smoking, household income, marital status, oral antidiabetic medication, insulin/other injectable diabetic medication, and time of testing. Regression models were repeated with the inclusion of daily mean sadness and significant results were maintained (baseline: *B* = −0.145, *p* = .015; immediately post-task: *B* = −0.149, *p* = .006; 45 min post-task: *B* = −0.143, *p* = .018; and 75 min post-task: *B* = −0.153, *p* = .007, see Table 3). Happiness was not significantly related to IL-6 change scores (*B* values between −0.022 and −0.011, and *p* ≥ .410) indicating no associations with IL-6 stress responses.

**Table 3.** Multiple regressions on daily happiness predicting baseline and post-task IL-6 (ln) after adjusting for covariates

	*B*	SE	ß	95% CI	*p*-value
Baseline IL-6 (*N* = 111)					
Happiness	−0.145	0.058	−0.226	−0.261 to −0.029	**.015**
Age	0.004	0.007	0.053	−0.010 to 0.019	.571
Sex	−0.192	0.099	−0.165	−0.389 to 0.004	.055
BMI	0.044	0.010	0.440	0.025 to 0.063	<.001
Smoking	0.271	0.138	0.175	−0.003 to 0.545	.053
Household income	−0.064	0.046	−0.135	−0.154 to 0.027	.166
Marital status	0.024	0.062	0.037	−0.098 to 0.146	.698
Oral antidiabetic medication	0.048	0.117	0.035	−0.184 to 0.279	.683
Insulin/other injectable diabetic medication	−0.035	0.150	−0.020	−0.333 to 0.262	.813
Time of testing	0.144	0.096	0.128	−0.047 to 0.335	.138
Sadness	−0.103	0.069	−0.138	−0.239 to 0.034	.140
Immediately post-task (*N* = 108)					
Happiness	−0.149	0.053	−0.241	−0.254 to −0.043	**.006**
Age	0.008	0.007	0.110	−0.005 to 0.021	.213
Sex	−0.094	0.089	−0.084	−0.272 to 0.083	.296
BMI	0.052	0.009	0.534	0.035 to 0.069	<.001
Smoking	0.278	0.128	0.183	0.024 to 0.531	.032
Household income	−0.074	0.042	−0.160	−0.157 to 0.010	.084
Marital status	−0.041	0.056	-0.065	−0.153 to 0.070	.465
Oral antidiabetic medication	0.086	0.105	0.066	−0.122 to 0.294	.412
Insulin/other injectable diabetic medication	−0.012	0.133	−0.007	−0.277 to 0.253	.927
Time of testing	0.256	0.087	0.236	0.083 to 0.429	.004
Sadness	−0.150	0.065	−0.201	−0.279 to −0.020	.025
45 min post-task (*N* = 99)					
Happiness	−0.143	0.060	−0.230	−0.261 to −0.025	**.018**
Age	0.008	0.008	0.099	−0.007 to 0.023	.318
Sex	−0.026	0.099	−0.023	0.222 to 0.171	.795
BMI	0.047	0.010	0.459	0.027 to 0.066	<.001
Smoking	0.208	0.137	0.141	−0.065 to 0.481	.134
Household income	−0.097	0.046	−0.212	−0.189 to −0.006	.038
Marital status	0.047	0.062	−0.076	−0.170 to 0.075	.446
Oral antidiabetic medication	0.059	0.113	0.047	−0.165 to 0.283	.601
Insulin/other injectable diabetic medication	−0.008	0.154	−0.005	−0.314 to 0.298	.957
Time of testing	0.245	0.097	0.226	0.052 to 0.438	.014
Sadness	−0.073	0.075	−0.098	−0.223 to 0.077	.333
75 min post-task (*N* = 95)					
Happiness	−0.153	0.056	−0.263	−0.264 to −0.042	**.007**
Age	0.005	0.007	0.068	−0.009 to 0.018	.494
Sex	−0.021	0.091	−0.020	−0.202 to 0.160	.819
BMI	0.048	0.009	0.480	0.029 to 0.066	<.001
Smoking	0.235	0.124	0.178	−0.012 to 0.481	.061
Household income	−0.067	0.043	−0.162	−0.153 to 0.018	.120
Marital status	−0.059	0.057	−0.103	−0.172 to 0.054	.305
Oral antidiabetic medication	0.108	0.101	0.095	−0.094 to 0.309	.291
Insulin/other injectable diabetic medication	0.018	0.143	0.011	−0.265 to 0.302	.899
Time of testing	0.245	0.088	0.247	0.070 to 0.421	.007
Sadness	−0.170	0.069	−0.248	−0.306 to 0.033	.016

*B* unstandardized beta coefficient; *CI* confidence interval; *IL-6* interleukin-6; *ln* log n; *ß* standardized beta coefficient; *BMI* body mass index; *SE* standard error. Bold values indicate statistically significant.

#### IL-1Ra

We did not find any associations between happiness and IL-1Ra before or after stress tasks (*B* values between −0.043 and −0.025, and *p* ≥ .352). There were also no significant associations between happiness and IL-1Ra stress responses (*B* values between −0.027 and −0.013, and *p* ≥ .072).

#### MCP-1

Participants with higher happiness score had significantly lower baseline MCP-1 concentrations (*B* = −7.815, *p* = .041) independently of age, sex, BMI, smoking, household income, marital status, oral antidiabetic medication, insulin/other injectable diabetic medication, and time of testing (Table 4). The associations between happiness and MCP-1 levels post-task were not significant (immediately post-task: *B* = −8.172, *p =* .062; 45 min post-task: *B* = 5.819, *p* = .172; and 75 min post-task: *B* = 5.853, *p* = .141). After the inclusion of daily mean sadness, results for baseline MCP-1 were rendered nonsignificant (*B* = −6.311, *p* = .131). There were no significant associations between happiness and MCP-1 stress responses (*B* values between 1.047 and 0.170, and *p* ≥ .606).

**Table 4:** Multiple regression on daily happiness predicting baseline MCP-1 after adjusting for covariates (*N* = 117)

	*B*	SE	ß	95% CI	*p*-value
Happiness	−7.815	3.782	−0.193	−15.314 to −0.316	**.041**
Age	0.890	0.486	0.180	−0.073 to 1.853	.070
Sex	−2.955	6.845	−0.040	−16.526 to 10.616	.667
BMI	0.894	0.654	0.141	−0.403 to 2.191	.175
Smoking	8.190	9.487	0.084	−10.619 to 26.998	.390
Household income	−2.646	3.126	−0.088	−8.843 to 3.551	.399
Marital status	1.964	4.225	0.048	−6.412 to 10.340	.643
Oral antidiabetic medication	−4.719	8.121	−0.055	−20.819 to −11.381	.562
Insulin/other injectable medication	4.241	9.865	0.040	−15.318 to 23.800	.688
Time of testing	3.579	6.578	0.51	−9.463 to −16.621	.587

*B* unstandardized beta coefficient; CI confidence interval; MCP-1 monocyte chemoattractant protein-1; *ß* standardized beta coefficient; *BMI* body mass index; *SE* standard error. Bold values indicate statistically significant.

## Discussion

We investigated the relationship between daily happiness and three inflammatory markers at resting and in response to mental stress tasks in people with T2D. The two tasks elicited significant increases in subjective stress and in IL-6. IL-6 was not significantly different from baseline until 75 min post-task, in agreement with the evidence of a relatively delayed IL-6 stress responsivity [18]. Interestingly, the stress of the tasks did not translate into significant increases in IL-1Ra or MCP-1 in people with diabetes, as observed in healthy samples [9, 18, 19]. One possibility is that the type of tasks or the timing of post-stress sampling played a role. In a previous study, IL-1Ra reached a peak at 90 min following social stress test [54]. In addition, in another study IL-1Ra showed a significant increase only at 120 min post-task [55]. IL-6 is one of the most frequent measured markers in laboratory studies as it is most consistently responsive to acute stress [18], and our findings add to the value of measuring IL-6 stress responsivity in people with T2D.

We hypothesized that participants with greater daily happiness would have lower IL-6, IL-1Ra, and MCP-1 concentrations at baseline and after stress, and smaller inflammatory stress responses, independently of covariates including daily sadness. Although we did not find any differential response to stress in people varying in happiness over the day, daily happiness predicted significantly lower IL-6 at baseline and all later time points, and lower baseline MCP-1. These effects were independent of age, sex, BMI, smoking, household income, marital status, oral antidiabetic medication, insulin/other injectable diabetic medication, and time of testing. The association between happiness and IL-6 remained after further controlling for daily sadness.

Previous studies of healthy individuals and people with heart failure recorded lower IL-6 concentrations in those reporting greater positive affect, but samples had only been analyzed under resting conditions [30–34]. The present results indicate that similar patterns exist in people with T2D. However, we did not observe differential inflammatory responses to stress in relation to happiness. This contrasts with our previous findings that IL-6 responses are reduced in more optimistic individuals [41]. In our study, IL-6 levels increased in parallel in people reporting higher and lower happiness. Consequently, absolute levels of IL-6 were greater in less happy people both before and after stress testing. The difference from previous studies involving healthy individuals may be related to the dysregulation of inflammatory, cardiovascular, metabolic, and neuroendocrine systems observed in T2D. Specifically, we recently found that people with T2D have increased basal IL-6 levels and show smaller IL-6 stress responses, compared with a healthy control group [22]. In the same study, people with diabetes showed smaller responses in systolic blood pressure, cortisol, and cholesterol, along with delayed recovery in systolic and diastolic blood pressure, cholesterol, and heart rate [22]. These physiological disturbances across multiple systems are indicative of heightened allostatic load in people with T2D, and might have accounted for the null findings on happiness and IL-6 stress responses in the current study.

Greater happiness was associated with lower baseline MCP-1. These findings add to the evidence that MCP-1 is sensitive to psychosocial factors [19, 37–39]. Contrary to our hypothesis, we did not find significant association between happiness and baseline IL-1Ra. Nevertheless, happiness represents only one dimension of subjective well-being, and a previous study has shown that in patients with heart failure, different positive affect measures are associated with different markers of inflammation [34]. Future research is needed to investigate associations between distinct positive constructs and different inflammatory markers in people with T2D, as well as their prospective association with objective health outcomes. We did not observe significant relationships between happiness and MCP-1 or IL-1Ra post-stress, or stress responses. These markers did not increase in response to the tasks, contrary to IL-6. Future studies need to replicate findings for IL-1Ra and MCP-1 in people with T2D.

Happiness was associated with lower IL-6 concentrations independently of negative mood, in line with previous studies [30, 34]. These findings suggest that the presence of happiness per se rather than the absence of sadness is associated with lower IL-6. Happiness may reflect a unique component of psychobiological processes taking place in people with T2D, and may be a better predictor of basal inflammatory function than neutral or sad affect.

The pathways linking happiness with lower circulating inflammatory factors are unclear. Nevertheless, ample evidence supports a bidirectional relationship between mood and inflammatory markers. For example, in both animal and human studies, the administration of pro-inflammatory cytokines increased the incidence of depressive symptoms [56]. Pre-clinical evidence shows that cytokines, including IL-6, act centrally to induce mood symptoms [56]. Therefore, lower basal inflammatory factors may have contributed to greater happiness. On the other hand, a number of laboratory studies demonstrated that psychological stress can induce an inflammatory response [9, 18]. Happier individuals may use more adaptive coping mechanisms when faced with daily stressors, resulting in a better basal activity of the stress-related systems, including the immune system.

### Clinical Implications

The lower IL-6 recorded in happier individuals with diabetes might contribute to reduced progression of diabetes in processes such as insulin resistance and dyslipidemia. High IL-6 concentrations inhibit AMP-activated protein kinase, an enzyme related in fatty acid oxidation, down-regulating the expression of genes involved in insulin-stimulated glucose transport and lipid uptake in adipose tissue [21]. Heightened IL-6 has been associated with new onset T2D in prospective [20] and cross-sectional studies [21], and pro-inflammatory states in people with the condition have been associated with macrovascular complications [28, 57].

Happiness might also contribute to a lower risk of cardiovascular disease (CVD) in people with T2D through reduced inflammatory factors. Elevated IL-6 concentrations are predictive of future CVD [23, 58] and poorer outcomes in people with established CVD [59]. In the ADVANCE trial of 3,865 people with T2D and established CVD risk factors, IL-6 was associated with future fatal and non-fatal cardiovascular events, after adjusting for other inflammatory markers including fibrinogen and C-reactive protein (CRP [28]). Moreover, two large-scale genetic studies provided evidence for a causal relationship between the IL-6 pathway and CHD. More precisely, it was found that the Asp358Ala variant in the IL-6 receptor gene, which attenuates IL-6 signaling on hepatocytes, monocytes, and macrophages, was associated with reduced production of CRP and fibrinogen, and subsequently, a reduced risk of CHD [60, 61]. Moreover, human and animal investigations provide evidence that MCP-1 is involved in the development of CVD [62], since heightened MCP-1 has been associated with subclinical atherosclerosis and newly diagnosed CHD [25]. Elevated MCP-1 levels may contribute to the pathogenesis of vascular diseases by enhancing recruitment of leukocytes to sites of inflammation to the vessel wall [63]. Another potential molecular mechanism is that MCP-1-induced protein (MCPIP), a novel zinc-finger protein, promotes oxidative and nitrosative stress that causes endoplasmic reticulum stress which leads to autophagy and cardiac cell death involved in heart failure [62].

Given that inflammatory markers are adversely related to diabetes pathology, positive psychology interventions to improve feelings of happiness are likely to be beneficial for this population, reflecting physiological outcomes important to health. A small, randomized trial targeting positive emotions in adults with T2D demonstrated a significant effect in reducing depressive symptoms in the intervention group [64]. Also, previous studies in patients with other chronic conditions showed that positive psychology interventions can lead to improvements in health behaviors and medication adherence [6]. This offers the possibility that these interventions in patients with T2D could also have an effect on behavioral adherence. Interestingly though, happiness was not related to diabetes control in this study, measured by HbA1c. Participants with higher HbA1c could be expected to be less happy, but people reported being happy independently of their glycemic control. Further studies are needed to confirm the efficacy of positive psychology interventions in people with diabetes and the mediating pathways.

### Study Strengths and Limitations

We deliberately recorded mood ratings repeatedly over several days so as to obtain an estimate of average happiness that was not biased by transient moods on a single occasion. We examined three inflammatory markers and their patterns of stress responsivity using a standard stress protocol. Although the study includes a relatively large sample of adults with diabetes, we were only able to collect full biological data on about three quarters of participants due to difficulties in blood sampling. The present study is also limited by the fact that laboratory responses were only tested on one occasion, and causal relationships between greater happiness in daily life and lower inflammatory markers cannot be drawn. Future studies need to shed light on the prospective association between happiness, inflammatory markers, and objective health outcomes in people with T2D. We included a long post-task blood sampling period. Nevertheless, an extended sampling phase could have provided additional information. Participants of this study were middle-aged men and women with T2D and without a history of CHD. They were recruited from the London area and most of them were of White European ethnicity, thus we do not know how far the results generalize to other cohorts.

## Conclusions

Lower baseline and post-stress IL-6 and lower baseline MCP-1 concentrations were observed in happier participants with T2D, independent of covariates. Significant associations for IL-6 were maintained after controlling for daily sadness. These findings suggest that greater happiness in daily life is related to decreased circulating inflammatory factors in adults with T2D, and that the effect of happiness is above and beyond the absence of sadness. Happiness in people with diabetes may contribute to better physical health through reduced inflammatory markers, but longitudinal studies are needed to confirm this protective pathway.
